# Whey: The Soil Bio-Community Enhancer That Selectively Controls Root-Knot Nematodes

**DOI:** 10.3390/plants8110445

**Published:** 2019-10-23

**Authors:** Nikoletta Ntalli, Maria A. Tsiafouli, Kaliopi Tzani, Olga Mavridi, Chrisostomos Oplos, Urania Menkissoglu-Spiroudi, Nikolaos Monokrousos

**Affiliations:** 1 Benaki Phytopathological Institute, 8 S. Delta Str., Department of Pesticides’ Control and Phytopharmacy, 14561 Athens, Greece; 2 Department of Ecology, School of Biology, Aristotle University, 54124 Thessaloniki, Greece; 3 Department of Soil Science of Athens, Institute of Soil and Water Resources, Hellenic Agricultural Organization- DEMETER, 14123 Athens, Greece; 4 Pesticide Science Laboratory, School of Agriculture, Faculty of Agriculture Forestry and Natural Environment, Aristotle University of Thessaloniki, 54124 Thessaloniki, Greece; 5 School of Science & Technology, International Hellenic University, 57001 Thessaloniki, Greece

**Keywords:** *Meloidogyne* spp., soil microbes, soil free-living nematodes, soil enzyme activities

## Abstract

To date, it is mandatory for ecofriendly pest-management tools to be used in agriculture. Whey is a dairy-processing waste, a plant and soil chemical and fungicidal basic substance. The beneficial effect of whey on soil microorganisms, enzymatic activities, and free-living nematodes—combined with its toxic activity on the plant parasites—forms root knot nematodes. In this study, this finding is reported for the first time. A drip-irrigating tomato plant combined with whey in water at 3.125% (*v/w*) and 6.25% (*v/w*) dose dependently promoted Gram+ and Gram− bacteria, actinomycetes, and fungi biomass. Respectively, whey treatment and duration augmented the bacterial feeding nematodes along with the soil enzymatic activities, e.g., alkaline phosphatase, dehydrogenase, and urease. The counterpart for these soil organisms’ and enzymes’ functionality is the decomposition of organic matter, nutrient mineralization and cycling. Additionally, whey applied at 6.25% (*v/w*) every 10 days in a field experiment exhibited an efficacy of 70% on root knot nematodes. It is calculated that the EC_50/3d_ value paralyzes in vitro *Meloidogyne javanica*, which was 3.2% (*v/v*). Conclusively, the soil application of whey could be a sustainable and ecofriendly method to combat the root knot nematodes and additionally to enhance soil biotic components.

## 1. Introduction

Whey forms as the result of liquid residue in cheese production, followed by milk curdle and strain. Dairy processing may produce 10 liters of whey per liter of processed milk, depending on the end product [1]. This leads to large amounts of industrial waste, despite its potential beneficial agricultural use. Many studies have focused on the direct effect of whey on plant growth, wherein researchers examined the effect of its application on several parameters regarding plant productivity and soil chemical properties. These studies concluded that whey can be used as a plant booster [2,3]. Even though these studies have focused on soil properties and plant growth, the effects of whey on various soil communities and free-living nematodes are yet to be investigated. Soil organisms such as bacteria, fungi, and nematodes are the main counterparts of significant soil processes like decomposition of organic residues, nutrient mineralization, and cycling [4,5,6]. In addition, soil enzymes contribute to the decomposition of organic matter and nutrient cycling. This is because they are parts of N, P, and carbon transformation [4,7]. Moreover, soil enzymes respond to soil management changes earlier than other soil-quality indicator changes. Therefore, it is of crucial importance to understand the effect of whey addition, diversity, and activity of soil organisms, as this might have an implication on soil functioning and plant performance.

Since 8 March 2016, whey has been considered a basic fungicidal substance for powdery mildews like *Podosphaera fusca*, *Podosphaera xanthii*, *Erysiphe cichoracearum, Erysiphe orontii, Sphaerotheca fuliginea*, and *Leveillula cucurbitacearum* [8]. Moreover, whey is reported to successfully reduce the wilt disease triggered by *Verticillium dahlia* in solanaceous crops [9]. Such root rot fungi usually form complexes in soil with the root knot nematodes *Meloidogyne*, a notorious agricultural pest known for its worldwide impact [10]. Traditionally, nematodes were controlled with chemical nematicides, many of which were globally banned because they were harmful to soil microbiota, animals, and humans [11]. However, the subsequent repetitive use of the few remaining commercial nematicides has led to detoxification mechanisms in soils and low efficacy under field conditions [12]. Alternative plant protection products—commonly known as biopesticides—are ecofriendly contrary to their synthetic ancestors. For this reason, they have been the focus of development [13]. Villaverde et al. reviewed the legislative framework of the use of natural products in an integrated crop-management scheme along with the mode of action and chemistry [14]. Bionematicides were reported to display complex modes of activity based on: i) direct lethal or sub-lethal properties; ii) elevation of plant growth and injury tolerance; and/or iii) inducing the interaction between the host and the nematode by influencing plant defenses [15]. Whey has been proposed as a soil treatment that could enhance crop growth and production [2,3], but to the best of our knowledge, bionematicidal properties against *Meloidogyne javanica* have yet to be tested.

In the present study, we assess the appropriateness of whey as both a soil enhancer and as a bionematicide against *Meloidogyne javanica.* We assess whey that has been applied on soil via drip irrigation in an effort to discern its effects on a single group of organisms or a single process, as well as on a broad spectrum of biotic soil attributes that cover the soil food web structure and functioning. Specifically, we quantified the short-term (15 days) and long-term effect (40 days) of whey in water (¼ and ½ *v/v*) applied with drip irrigation in soils that hosted tomato plants at the concentrations of C_25_: 3.125% (*v/w*) and C_50_: 6.25% (*v/w*) per soil unit. We consider: (a) microbial communities by analyzing the phospholipid fatty acid (PLFA) soil profiles; (b) soil nematode communities by analyzing their trophic, functional, and community structure; (c) the activities of urease, alkaline phosphatase, and dehydrogenase; and (d) plant growth regulation or phytotoxicity. Considering the use of whey as bionematicide, we evaluated its action as a paralysis agent against *M. javanica* J2. Furthermore, we tested this activity under open field conditions.

## 2. Results

**Figure 1:**
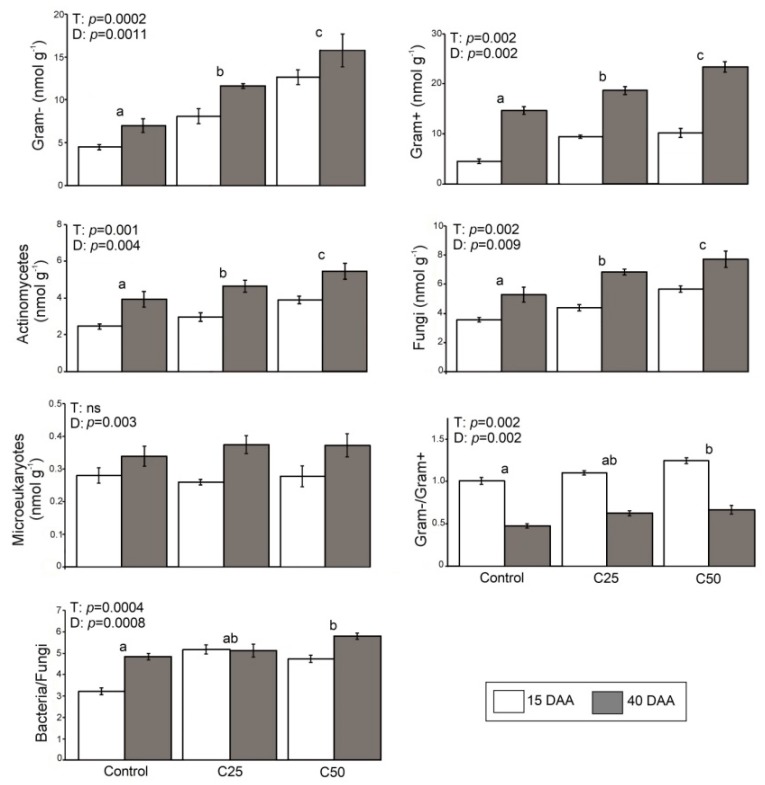
Mean values (± st. error) of variables for microbial groups and results of PERMANOVA regarding the effects of “treatment” (T) and “duration of treatment” (D) on these parameters. Different letters (a, b, c) indicate significant differences of pairwise comparisons among treatments (Control, C_25_ and C_50_).

**Figure 2:**
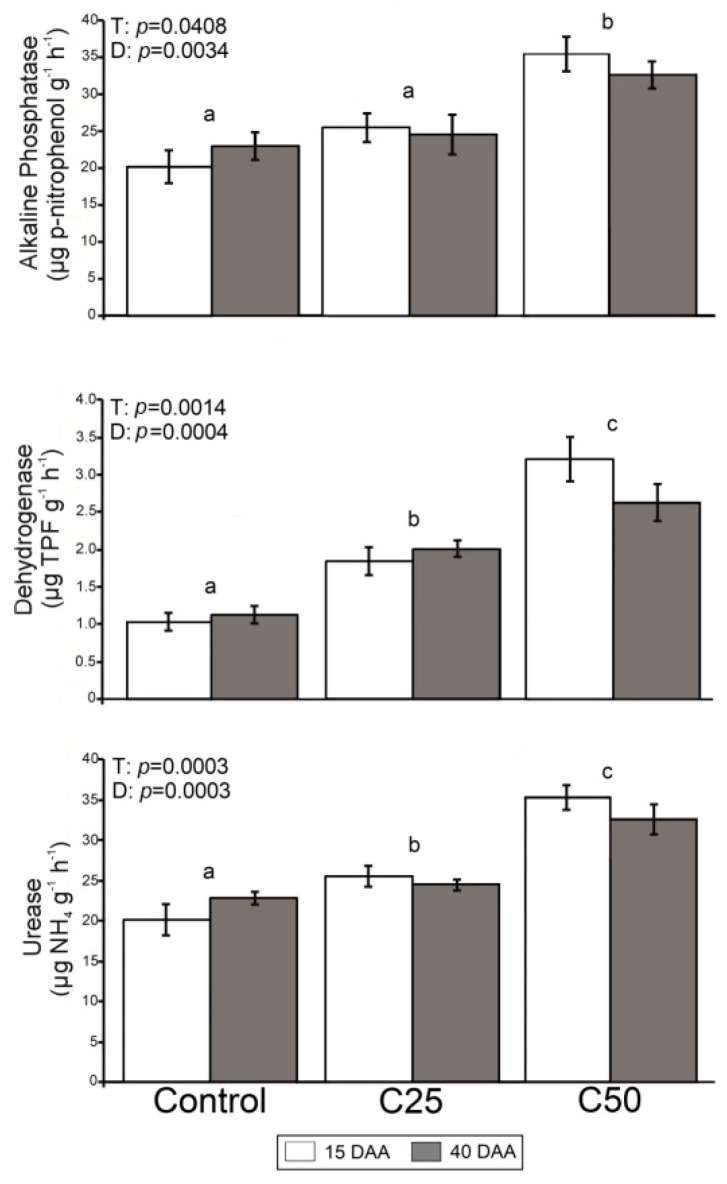
Mean values (± st. error) of soil enzyme activities and results of PERMANOVA regarding the effect of “treatment” (T), “duration of treatment” (D) on these parameters. Different letters (a, b, c) indicate significant differences of pairwise comparisons among treatments (Control, C_25_ and C_50_).

**Figure 3:**
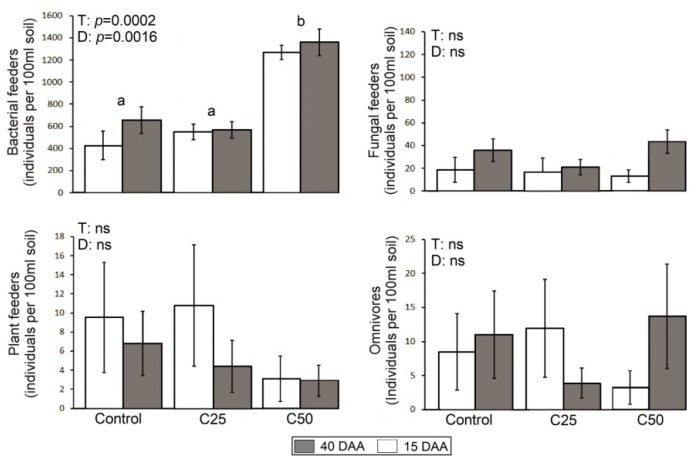
Mean values (± st. error) of variables for nematode trophic group abundances and results of PERMANOVA associated with “treatment” (T) and “duration of treatment” (D) on these parameters. Different letters (a, b) indicate significant differences of pairwise comparisons among treatments (Control, C_25_ and C_50_). The results of principal component analysis (PCA), depicting the ordination of samples and variables are presented in Figure 4. The first axis explained 54.4% of the data variability, while the second one explained 17.5% (71.9% in total). Samples of the C_50_ for both durations were ordinated at the left part of the first axis showing a positive correlation mostly to Gram−, Gram+, fungi, and bacterial-feeding nematodes. The samples of control and C_25_ of 15DAA were placed at the right side of the first axis showing a positive correlation to plant-feeding nematodes. Samples of 15DAA were separated along the second axis from all other samples and were ordinated in the lower part, showing a positive correlation to urease, dehydrogenase and alkaline phosphatase activities.

The mean value of microbial group variables, enzyme activities, and nematode abundances for the different treatments (C_50_, C_25_, control) and duration of treatment (15DAA and 40DAA) are given in Figure 1, Figure 2 and Figure 3, respectively. Results of PERMANOVA (Permutational Multivariate Analysis of Variance) regarding the effect of “treatment”, “duration of treatment” (within factor “treatment) on these parameters are shown in each figure separately.

### 2.1. Effects of Whey on the Microbial Communities

All microbial group abundances (Figure 1) were significantly affected by treatment and duration, with the exception of microeukaryotes abundance, which was only affected by duration. Gram+, Gram−, actinomycetes, and fungi values were significantly higher in the C_50_ and C_25_ treatments relative to the control. Values of the C_25_ treatment were significantly lower than those recorded in C_50_. Additionally, most microbial variables showed increased values in 40DAA compared to those in 15DAA. Regarding the bacteria/fungi and Gram-/Gram+ ratios, the C_50_ samples had significantly higher values than the control samples, while the 15DAA samples were lower compared to 40DAA for the bacteria/fungi and higher in the Gram^−^/Gram^+^ ratio.

### 2.2. Effects of Whey on Enzyme Activities

Treatment and duration significantly affected alkaline phosphatase, dehydrogenase and urease activities (Figure 2). C_50_ presented the most elevated values of all enzyme activities and differed significantly compared to the control. The C_25_ values were lower than C_50_, but were significantly higher in comparison to the control for dehydrogenase and urease. The effect “duration of treatment” was also significant but did not exhibit the same pattern for the treated and control samples. In the case of the latter all measured enzyme activities were lower in 15DAA compared to 40DAA, while the opposite was observed for C_50_ and C_25_ (except for dehydrogenase).

### 2.3. Effects of Whey on Nematode Bio-Communities

Regarding nematodes, four different trophic groups were found in the samples, namely fungal feeders, bacterial feeders, omnivores and plant feeders. We did not find any predatory nematodes. Bacterial feeding nematodes abundance (Figure 3) was the only trophic group that was significantly and positively affected by whey addition, exhibiting a threefold increase in the C_50_ 15 DAA and 40DAA. Plant feeders showed a decline in numbers in the C_50_ but this decline was not statistically significant as the numbers of individuals were very low also in the control samples (approximately 8 individuals/100 mL soil) and slight changes in numbers led to high variations between samples. The same held more or less for the trophic group of omnivores.

**Figure 5:**
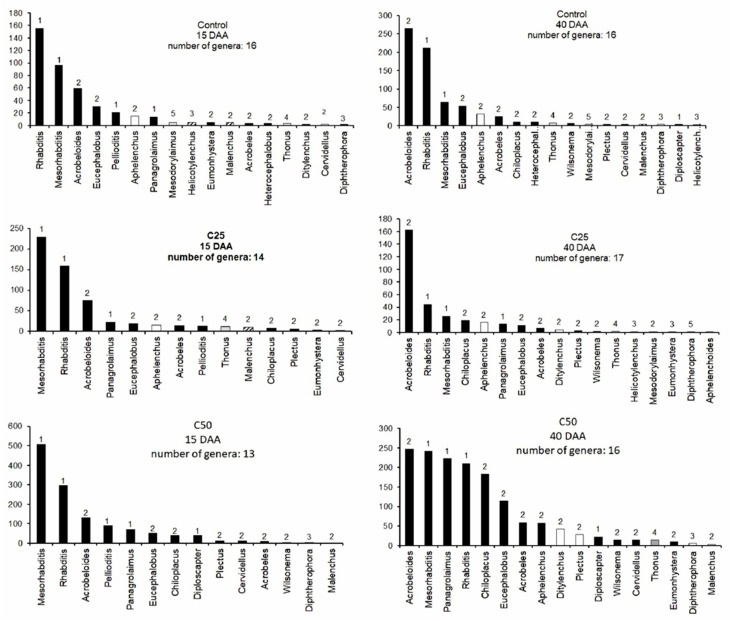
Rank abundance graphs for nematode genera in the community for each treatment in both sampling occasions. Numbers above the bars correspond to the c-p value of each genus. (Bar color: Black-bacterial feeders, White-fungal feeders, White with Diagonal pattern-plant feeders, Grey-omnivores).

The c–p value, the trophic type and the abundance of each nematode genus are presented in Figure 5 as rank abundance graphs for each treatment and both treatment durations; all treatments presented similar numbers of genera (13–17) while in 40DAA the numbers were found to be more elevated in the whey treatments, in comparison to those recorded 15DAA. In 15DAA all samples were characterized by the over-dominance of c-p1 bacterial-feeding nematodes, *Rhabditis* in the control and *Mesorhabditis* in C_50_ and C_25_ treatments. In 40DAA the pattern was different as in all treatments the c-p2 bacterivore nematode *Acrobeloides* was the dominant genus. Moreover, in the C_50_ treatment the nematode community was no longer characterized by the dominance of one single genus but a more equally distributed community was observed, involving c-p1 as well as c-p2 nematode genera (*Acrobeloides*, *Mesorhabditis*, *Rhabditis*, *Panagrolaimus*).

Regarding plant parameters no phytotoxicity was evident at either of the whey twofold test concentrations employed in the bioassay. Furthermore, no significant differences were observed amongst aerial part and root weights of tomato plants treated with whey (data not shown).

### 2.4. Paralysis Effect of Whey on the Plant Parasitic Nematode M. Javanica

According to the paralysis experiment, a dose-response relationship was evident and paralysis activity increased over time. In particular, one day after J2 dipping in test solutions the EC_50_ value was calculated at the dose of 4.9% (*v/v*), while after two days it decreased at the level of 3.2% (*v/v*) (Table 1). The nematicidal potential of whey cannot be attributed to lactic acid contents, since preliminary experiments showed no paralysis activity on J2 for test concentrations up to 1000 ppm (data not shown).

### 2.5. Field Experiment

**Figure 6:**
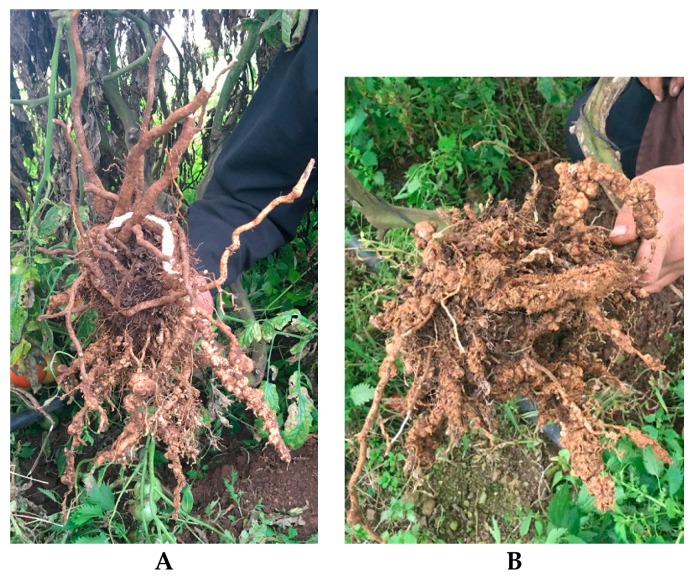
(**A**): Root of whey-treated tomato plant at the end of the culturing season, (**B**): Untreated control tomato root.

According to the galling index, all control plants were severely knotted (gall index 9–10) because of the secondary infections by soil pathogens, while in whey-treated plants only a few knots were formed (gall index 3) significantly lower to control (*p* < 0.05). Whey applied by drip irrigation under field conditions in 10 days interval till harvest controlled root knot nematodes at a percentage of 70% (Figure 6), while no differences were evident regarding the yield of tomato plants (data not shown).

## 3. Discussion

Our study showed that whey has multiple effects for soil biotic components, acting as both a soil foodweb enhancer and a nematicide affecting negatively the agricultural pest *M. javanica*. Whey contains soluble proteins, lactose, vitamins and minerals [2] that can be used as a carbon and nitrogen source by microorganisms [16,17]. Our study showed that the effect of whey is concentration-dependent, as both C_25_ and C_50_ treatments appeared to induce a significant increase in the abundance of all microbial groups (Gram+, Gram−, actinomycetes, fungi) compared to the control samples, while C_50_ presented significantly the highest values. Thus, whey additions have a positive impact on all groups in the soil microbial community. All 40 DAA treatments presented higher abundances of all estimated microbial groups, compared to those recorded for 15 DAA because as the tomato plants grow, the roots excrete larger amounts of organic substances for microbial growth [18,19,20]. Microeukaryotes comprised the only group that was not significantly altered by whey addition. This could be attributed to the fact that microfauna organisms have longer life cycles, exceeding the experiment’s duration [21].

In relation to microbial community structures, both whey treatments resulted in higher bacteria/fungi ratios compared to the control. However, this increase was more pronounced 15DAA, as the aforementioned whey labile organic substrates seem to favor the bacterial proliferation, giving them predominance over fungi [22]. Specifically, the addition of whey favors the growth of Gram– bacteria, which have higher adaptability to differentiated environmental circumstances [23], and thriving proliferation [24,25,26]. Thus, the higher the amount of whey added, the stronger the effect in the soil microbial community structure. At 40DAA, even though both bacterial and fungal abundances were further increased and bacteria maintained their dominance over fungi, the composition of the soil bacterial community was found to differ significantly to that recorded at 15DAA. The Gram−/ Gram+ ratio at 40DAA (in the control and in both treatments) was found to be lower compared to the one recorded at 15DAA, showing that the root growth over time favored the proliferation of Gram+ over Gram− bacteria. It seems that plant growth contributes to the homogenization of soil conditions, and promotes a more stable soil system, creating the proper conditions for Gram+ bacteria growth [20].

Whey had a positive effect on soil functionality considering the increase of the enzyme activities of dehydrogenase, urease and alkaline phosphatase. Since the soil enzyme activity is highly correlated to the microbial biomass [27], the elevated enzymatic activities recorded in the C_25_ and C_50_ treatments could be attributed to the increased amounts of C and N after the whey additions. Both C_25_ and C_50_ treatments increased the dehydrogenase and urease activity rapidly (15 DAA) until the end of the experiment. Dehydrogenase often represents a measure of the overall soil microbial activity [28,29] and changes in the metabolic state of soil microorganisms, providing a good index of soil quality [30]. On the other hand, urease is a counterpart in the soil–nitrogen cycle as it catalyzes the release of NH4^+^ from urea [31], is a constitutive–intracellular enzyme and, thus, it increases with microbial biomass [32]. It is possible that the high protein content of the whey provides nitrogen-rich substances to trigger the processes related to the cycle of nitrogen [33]. Last, the alkaline phosphate activity presented a slightly different pattern as its values in the C_25_ treatment did not differ significantly from the control. The alkaline phosphatase is mainly extracellular and is released by microorganisms in response to augmented substrates [34]. This could imply that only larger additions of whey affect the P cycle. Overall, the addition of whey in the C_50_ treatment proved to have a long-term result on all estimated soil-enzyme activities, and consequently to improve soil functionality.

In our study we found four major trophic groups of nematodes, namely bacterial feeders, fungal feeders, plant feeders and omnivores, the latter two groups in very low abundances. We did not find any predatory nematodes, which are usually of high longevity, large body size and highly sensitive to disturbance, indicating that the bio-community in our pot experiment had a very loose, disturbed community structure. This was probably a combined result of the origin of the soil (agricultural soil, historically disturbed) and of the disturbance of the soil for the preparation of the pot experiment. The bacterial-feeding nematodes were the prevailing trophic group in all treatments, including the control, and contributed in most cases over 90% of the entire community. The dominance of bacterivorous nematodes is expected for agricultural soils [35,36] and is the common case in pot experiments [37]. The incorporation of whey increased the abundance of bacterial feeders, but only in C_50_, where we observed two- or three-times higher abundances compared to the control in 15 and 40DAA. Mostly nematodes belonging to the c-p1 group, such as *Rhabditis* and *Mesorhabditis*, were those that increased in abundance (see Figure 5). Nematodes that belong to this group are considered enrichment opportunists as they respond positively and rapidly to disturbances that result in enrichment by nutrients and the bloom of bacteria [5,38,39,40]. Whey increases the C:N ratio in soils due to the high quantities of easily degradable carbon, increasing the numbers of bacteria [40]. Thus, it is evident that changes in the abundances of nematodes are following changes of their prey, i.e bacteria. The c-p1 bacterial feeders prefer to feed mostly on Gram– bacteria, rather than Gram+ bacteria due to certain physiological characteristics [41,42,43,44]. This is also supported also by our principal component analysis (PCA), where Gram− and bacterial feeders are grouped together joining also the enzymes urease, dehydrogenase and alkaline phosphatase. The addition of whey to the soil favored in general the bacterial decomposition pathway, but mostly those parts that are able to respond rapidly to changes.

Regarding the fungal decomposition pathway, although fungi showed an increase with the addition of whey, fungal feeding nematodes did not change accordingly. This might be attributed to the fact that their populations were very low in the control samples (on average 20–40 ind/100 mL soil). Regarding the plant-feeding nematodes, the genera *Malenchus* and *Τylenchus* found in our soil are characterized as plant non-parasitic feeders as they do not comprise economically important plant parasites [45]. No significant difference was recorded among treatments or duration of treatment for this group. Generally, the numbers of plant-feeding nematodes in our study were low, which might be attributed to the fact that the plants in the pots were small. It has been reported that in soils with low root biomass the plant-feeding nematode population is relatively low [46,47]. Even in 40DAA the plants in the pots did not grow enough to support a significant increased population of plant-feeding nematodes.

At 15DAA nematode genera of c-p1 were the most abundant in all treatments. The incorporation of whey in the soil consists of a positive disturbance that favors the increase of fast-growing decomposers of the soil food web (c-p1 nematodes, Gram− bacteria). Our results are in agreement with Ferris [38], who reported that the enrichment of soil with nutrients is expected to increase the opportunistic (c-p1) bacterial-feeding and fungal-feeding nematodes. After the enrichment phase faded out, the abundances of c-p1 bacterivore groups, that have primarily participated in the nutrient mineralization cycles, decreased while the numbers of the c-p2 functional guilds increased afterwards; 40DAA, genera belonging to the c-p2 group become dominant in all treatments, indicating the recovery of the nematode community from the disturbance [5,48]. C50 was the only treatment that was not characterized by the dominance of a single genus in the nematode community 40DAA. This result could suggest that the incorporation of greater amounts of whey in a soil system could result in greater diversity of nematode genera, creating a more complex food web network, and possibly a more stable ecosystem function [49].

Last, regarding the nematicidal activity of whey, some reports involve the activity of its proteins. In particular, larval immotility of *Ostertagia circumcincta*, a most important parasite of sheep and goats, increased in whey protein solutions from 5%–15%, from pH 2.5–6.5 and from 2 to 24 hours of immersion (all *p* < 0.001) [50]. In our study, a correlation dependency has been revealed between biological activity in terms of paralyzed J2 and the concentration of whey at the range of 2 to 5% (*v/v*). The established EC_50_ values of whey calculated against the phytoparasitic nematode *M. javanica* were close (3.2 to 4% *w/w* after 1 and 3d of immersion) to the concentrations of whey used for soil treatment (3.125 and 6.25% *v/w*) to promote soil bio-communities. Moreover, the in vitro efficacy results were confirmed under field conditions and whey achieved efficacy over 70% on tomato plants treated with whey at 6.25% *v/w* in soil. In conclusion, whey enhances soil bio-communities’ and controls substantially the root-knot nematodes under field conditions. The implications of our findings are promising for crop protection. Whey, in addition to its known fungicidal properties [8,51], gains the potential to be used in integrated nematode management programs.

## 4. Conclusions

To date only the use of whey as basic ingredient in fungicides has been approved. According to our findings whey exhibits nematicidal potential against *Meloidogyne javanica* without harming soil microorganisms and free-living nematodes. Thus, our findings underline whey’s potential in an integrated pest-management program.

## 5. Materials and Methods

### 5.1. Pot Bioassay

Whey produced from goat’s milk was obtained from a local cheese producer. The goats were not treated with antibiotics so as not to interfere with the results of the study. Soil was sampled from a greenhouse at Benaki Phytopathological Institute (Athens, Greece) and was partially dried overnight and sieved through a 2 mm mesh sieve to remove debris. We determined the organic matter, pH and soil texture using the methods of Karpouzas and co-workers [52]. The soil was characterized as a sandy loam (clay: 18%, silt: 22%, sand: 60%), with 3.3% organic carbon, 1.9 mg g^−1^ total N, and pH 6.5. The soil was mixed with sand at the ratio 2:1 (sand:soil). An amount of 200 gr soil was used for each pot and tomato plants cv. Belladonna, at the six-leaf stage (one plant per pot), were transplanted. Each pot received 25 mL of whey solution at the test concentrations of 50% *v/v* and 25% *v/v*. The dilutions were made with distilled water. Thus, the experimental treatments were C_25_: 3.125% (*v/w*) and C_50_: 6.25% (*v/w*) of pure whey volume in soil. Water was used as a control at the same final volume. The tomato plants were conserved at 27 °C, 60% RH and 16 h photoperiod. 25–30 mL of water were used per pot every 2 days for a total of 45 days. Care was given to avoid runoff. Two destructive samplings were performed on two assessment dates, namely 15 days after application (15DAA) and 40 days after application (40DAA). The treatments were arranged in a completely randomized design with five replicates. In each destructive sampling, for PLFA, enzymatic activities and soil nematodes analyses, a composite soil sample from each replicate of the pot treatments was taken. Enzyme activity assays were performed the same day, while samples for PLFA and free-living nematodes analysis were stored at 4 °C and analyzed within a week. Finally, the fresh weights of the roots and aboveground parts were measured.

#### 5.1.1. Phospholipid Fatty Acid (PLFA) Analysis

Soil samples were analysed for PLFA content according to the method described in detail by Ntalli and co-workers [37], as PLFA analysis is widely used to measure soil microbial biomass and community composition.

Overall, in all soil samples, we consistently found 25 PLFA biomarkers which were included in all further analyses. We assigned these PLFA biomarkers to functional groups as follows [26,53,54,55,56]: 15:0, i-15:0, a-15:0, i-16:0, 17:0, i-17:0, (Gram-positive bacteria); 16:1ω9c, 16:1ω9t cy17:0, (Gram-negative bacteria); 10Me16:0, 10Me17:0, 10Me18:0 (actinomycetes); 18:2ω9,12 (fungi); 20:0, 22:0, and 24:0 (microeukaryotes); 16:0 may derive from bacteria and fungi, 18:1ω9c and 18:1ω9t from both fungi and Gram-negative bacteria, while 13:0, 14:0, 17:1, 18:0, 18:2ω6t and 19:1ω9c are of microbial origin. Based on the above, we also estimated the Gram^+^/Gram^−^ and bacteria/fungi (B/F) ratios.

#### 5.1.2. Enzyme Activity Assays

The method suggested by Tabatabai [57] was used for the determination of the urease and alkaline phosphatase the activities. We estimated urease activity based on the amount of NH_4_^+^ that was released when soil was incubated at 37 °C for 2 h with 0.1 M Tris (hydroxymethyl) aminomethane buffer, toluene, and urea. The quantitate determination of released *p*-nitrophenol was used for the estimation of alkaline phosphatase activity, after the incubation of soil with *p*-nitrophenyl phosphate in modified universal buffer (pH = 11). Dehydrogenase activity (DHG) was estimated after the incubation of soil at 37 °C for 24 h with an aqueous 2,3,5-triphenyltetrazolium chloride solution and CaCO_3_, followed by spectrophotometric measurement of the amount of triphenylformazan (TPF) (formazan release method).

#### 5.1.3. Soil Nematodes Extraction of and Community Analysis

We used 100 mL of each composite soil sample for nematode extraction based on Cobb’s sieving and decanting method, as modified by S’Jacob and van Bezooijen [58]. Living nematode abundance was estimated under a stereoscope and thereafter nematodes were fixed in 4% formaldehyde. We used the identification key of Bongers [59] to identify 150 randomly selected nematode individuals to genus level, under the microscope, from each sample. We further assigned them to functional groups according to Ferris and co-workers [38] and trophic groups according to Yeates and co-workers [60].

### 5.2. Paralysis Bioassays Against Meloidogyne Javanica

A population of *M. javanica* was reared from a single egg mass on tomato (*Solanum lycopersicum* Mill.) cv. Belladonna. Freshly hatched (24 h) J2 were obtained from 60 d nematode-infested roots [61], to be used for the experiments. Whey was diluted in water to prepare test solutions, which were mixed in Cellstar 96-well cell culture plates (Greiner Bio-One) at a ratio of 1:1 (*v/v*) with nematodes’ suspension. Each well contained 15–20 J2s and the whey solutions in distilled water yielding the test concentrations of 2–5% (*v/v*). Border wells containing J2s immersed in water assisted for fumigant activity evaluation [62]. Multiwell plates were covered with lids and were preserved in the dark at 20 °C throughout the experiment’s duration. After 1d and 3d the juveniles were classified into moving and paralyzed, at 40× with the aid of an inverted microscope (Euromex, The Netherlands). After the last assessment, motility regain was checked by transferring J2 to tap water and observing again after 24 h. In all cases neither activity regain nor fumigant activity was evident. Treatments of paralysis experiments were replicated six times, and each experiment was performed twice.

### 5.3. Field-Efficacy Experiment against the Root Knot Nematodes

To confirm the efficacy in field conditions, whey was used by drip irrigation on tomatoes cv. Belladonna naturally infested with root knot nematodes. Specifically, the trial consisted of two treatments, namely (1) tomato plants treated with C_50_: 6.25% (*v/w*) whey every 10 days till harvest and (2) an untreated control drip irrigated with water. Each treatment had four replicates, and each plot area was 10 m^2^. During the culture season there were no other applications for nematode control, and the efficacy was evaluated 73 days after transplanting using the root index. Additionally, the weight of roots and aerial parts was recorded.

### 5.4. Statistical Analysis

#### 5.4.1. Testing Effects of Whey as a Soil Amendment

For each individual response variable regarding microbes (abundances and ratios), enzyme activities and nematodes (abundances), we evaluated the effects of Treatment (C_50_, C_25_, control) and duration of treatment (15DAA, 40DAA) by the application of permutational analyses of variance to our data. All PERMANOVA analyses were performed with treatment as fixed factor and duration nested within treatment. For all cases we used 4999 permutations and the deviance of dissimilarities was the distance measure to generate dissimilarity matrices for the data. Pair-wise a posteriori tests were performed among levels of factor: (a) treatment, (b) duration of treatment where feasible. For these analyses the Fortran software PERMANOVA [63] was used.

A Principal Component Analysis (PCA) was applied to our data, in order to explore whether treatment or duration of treatment is more significant for the variability presented by the entire dataset. Τhe ordination of the corresponding samples was done according to the generic structure of the nematode communities, the structure of microbial communities and enzyme activity variables.

We constructed rank abundance graphs showing the contribution of each nematode genus its “Colonizer-Persister” (c-p) value and trophic group. The c-p value (1–5) is analogous to the r-K strategy continuum, where the c-p1 nematodes are r-strategist and the nematodes with c-ps > 3 have characteristics closer to the K strategists [64].

#### 5.4.2. Paralysis Effects of Whey on *Meloidogyne javanica* and Field-Efficacy Experiment

J2 paralysis results were statistically elaborated according to Ntalli and co-workers to calculate EC_50_ values [62]. Briefly, the percentages of paralyzed J2 witnessed in the microwell assays on each assessment date were corrected by excluding the natural death according to the Schneider Orelli’s formula and they were analyzed (analysis of variance, ANOVA) after being combined over time [65]. Since ANOVA indicated no significant treatment by time interaction, means were averaged over experiments. Corrected paralyses was subjected to nonlinear regression analysis using the log-logistic equation proposed by Seefeldt and co-workers [66] Galling index and tomatoes production were subjected to Tukey’s test to separate treatment differences, *p* ≤ 0.05. Because ANOVA indicated no significant treatment by time interaction (between runs of experiments), means were averaged over experiments.

## Figures and Tables

**Figure 4:**
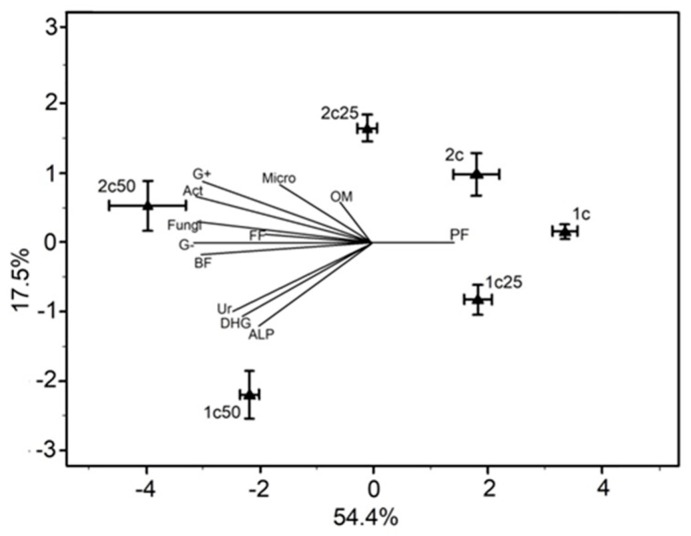
Ordination of the soil samples on a PCA biplot based on the generic structure of nematode communities, the enzyme activities and the microbial group variables. Each point corresponds to a sample collected at a specific time (1:15DAA, 2:40DAA), and from a specific treatment (Control, C_25_, C_50_). Error bars indicate standard errors.

**Table 1:** EC_50_ (% *w/v*) values of cheese whey against *M. javanica* calculated after immersion of J2s in test solutions for 1 and 3 days.

Days of Exposure	EC_50 (% *v/v*)_	Std. Error	CI_95_%
1	4.9	0.602	3.6–6.1
3	3.2	0.137	2.9–3.5

## Abbreviations

c-p	Colonizer-Persister
C_25_	3.125% (*v/w*) of whey in soil
C_50_	6.25% (*v/w*) of whey in soil
EC_50/3d_	half maximal Effective Concentration after 3 days
